# Atomic layer deposition of dielectric Y_2_O_3_ thin films from a homoleptic yttrium formamidinate precursor and water†

**DOI:** 10.1039/d0ra09876k

**Published:** 2021-01-12

**Authors:** Nils Boysen, David Zanders, Thomas Berning, Sebastian M. J. Beer, Detlef Rogalla, Claudia Bock, Anjana Devi

**Affiliations:** Inorganic Materials Chemistry, Ruhr University Bochum 44801 Bochum Germany anjana.devi@rub.de; Microsystems Technology, Ruhr University Bochum 44801 Bochum Germany; RUBION, Ruhr University Bochum 44801 Bochum Germany

## Abstract

We report the application of tris(*N*,*N*′-diisopropyl-formamidinato)yttrium(iii) [Y(DPfAMD)_3_] as a promising precursor in a water-assisted thermal atomic layer deposition (ALD) process for the fabrication of high quality Y_2_O_3_ thin films in a wide temperature range of 150 °C to 325 °C. This precursor exhibits distinct advantages such as improved chemical and thermal stability over the existing Y_2_O_3_ ALD precursors including the homoleptic and closely related yttrium tris-amidinate [Y(DPAMD)_3_] and tris-guanidinate [Y(DPDMG)_3_], leading to excellent thin film characteristics. Smooth, homogeneous, and polycrystalline (fcc) Y_2_O_3_ thin films were deposited at 300 °C with a growth rate of 1.36 Å per cycle. At this temperature, contamination levels of C and N were under the detectable limits of nuclear reaction analysis (NRA), while X-ray photoelectron spectroscopy (XPS) measurements confirmed the high purity and stoichiometry of the thin films. From the electrical characterization of metal–insulator–semiconductor (MIS) devices, a permittivity of 13.9 at 1 MHz could be obtained, while the electric breakdown field is in the range of 4.2 and 6.1 MV cm^−1^. Furthermore, an interface trap density of 1.25 × 10^11^ cm^−2^ and low leakage current density around 10^−7^ A cm^−2^ at 2 MV cm^−1^ are determined, which satisfies the requirements of gate oxides for complementary metal-oxide-semiconductor (CMOS) based applications.

## Abstract 2

In this work, the application of tris(*N*,*N*′-diisopropyl-formamidinato)yttrium(iii) [Y(DPfAMD)_3_] as a precursor in a water-assisted thermal atomic layer deposition (ALD) process for the fabrication of device quality Y_2_O_3_ thin films is demonstrated.

## Graphical abstract

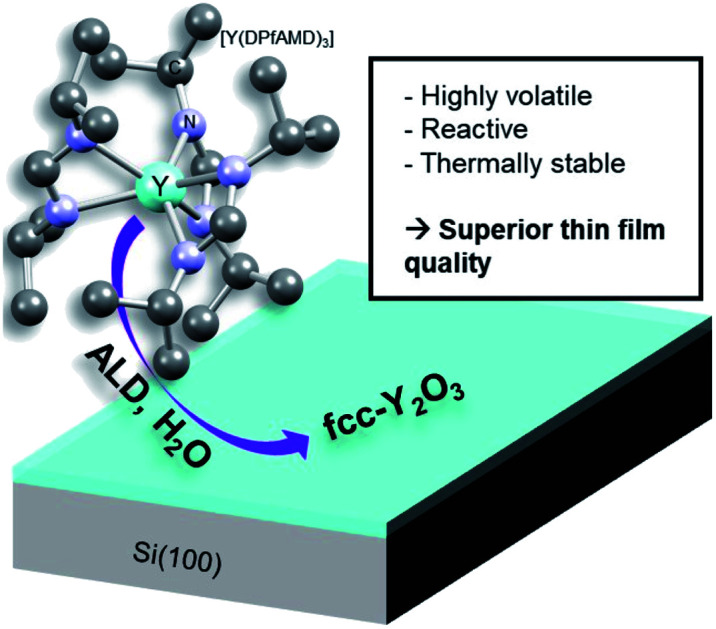

## Introduction

**Fig. 1:**
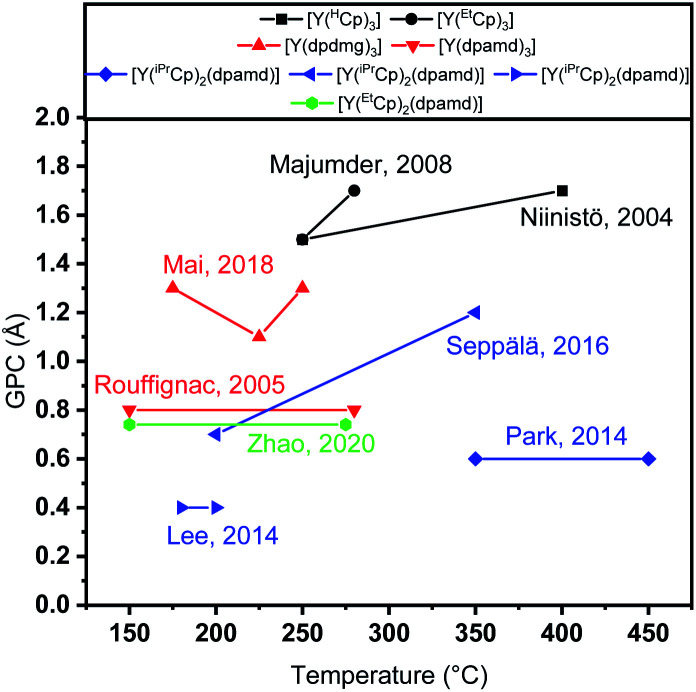
Representation of the water-assisted thermal ALD processes employing different precursors. Black: cyclopentadienyls, red: homoleptic yttrium amidinates and guanidinates, blue: heteroleptic yttrium isopropyl-cyclopentadienyl amidinates, green: heteroleptic yttrium ethyl-cyclopentadienyl amidinates.^12–15,17–20^

Yttrium(iii) oxide (Y_2_O_3_) thin films play an important and versatile role in various applications which arise from the valuable intrinsic material properties: the high relative permittivity (*e*_r_ = 14–18) and a large direct band gap (*E*_g_ = 5.5–5.8 eV) render this material useful as a high-*κ* material for its implementation as a potential gate dielectric in metal oxide semiconductor field effect transistors (MOSFETs).^1,2^ Additionally it features an intrinsic hydrophobic surface owing to its special electronic structure among the other rare-earth oxides,^3^ which together with its high chemical resistivity,^4^ mechanical strength and melting point render Y_2_O_3_ a useful candidate for application as protective coating even in very harsh environments.^5,6^ Moreover, a high refractive index of *n* = 2.1 enables the application of Y_2_O_3_ as a waveguide in solid state lasers.^7,8^ For all these applications, it is desirable that the coating is thin, while retaining its valuable intrinsic properties. Vapour phase depositions of thin layers of Y_2_O_3_ on complex substrates, such as three-dimensional or sensitive surfaces is conveniently realized by atomic layer deposition (ALD). This technique enables the growth of a variety of materials with high degree of homogeneity, good compositional control and conformality due to controlled layer-by-layer growth.^9^ Such a growth is initiated by saturative adsorption and reaction of the employed precursor on the surface of the substrate. The chemistry of the precursor and co-reactants have a significant influence on growth rate (growth-per-cycle, GPC), composition, structure and morphology of the thin film.^10^ In an ideal case, the growth rate within the so-called ALD window is independent of the deposition temperature, which in reality however is not a necessity to obtain high quality thin films.^11^ It should be noted that not only the growth rate, but also the chemical and physical quality of the resulting thin films mainly dictate how broad or narrow the ALD window can be considered and thus in which range a high thin film quality can be retained. In general, a broad ALD window can be achieved if the volatility and reactivity of the precursor is sufficient to prevent condensation and ensure chemisorption on the substrate at lower deposition temperatures, while the thermal stability and adsorption strength of the precursor prevents decomposition or desorption processes at higher temperatures. A rational choice of the ligands is a crucial step in the successful development of precursors to avoid thermal decomposition and ensure a high reactivity and volatility. For instance, incremental changes within the chemical backbone of the ligands can have a considerable influence on the physicochemical properties of the precursors, ALD process parameters and quality of the resulting thin films. In the past, different ALD precursors for the deposition of Y_2_O_3_ have been employed. Especially the precursors based on the yttrium cyclopentadienyls [Y(Cp)_3_] clearly demonstrate how small changes in the substitution pattern of the ligand can influence the ALD relevant properties of the complexes, which has a strong influence on their behaviour in the respective ALD processes (Fig. 1).^12,13^ Exemplarily for water-assisted ALD processes, the cyclopentadienyl based complexes, [Y(^H^Cp)_3_] and [Y(^Et^Cp)_3_] show a similar growth rate in the range of 1.5–1.7 Å per cycle, whereas the reported ALD process window is higher for [Y(^H^Cp)_3_] with a maximum deposition temperature of 400 °C. On the contrary, the volatility of [Y(^Et^Cp)_3_] is higher which enables the use of lower bubbler temperatures (150 °C for [Y(^H^Cp)_3_] and 120 °C for [Y(^Et^Cp)_3_]), while carbon contamination in the thin films is reported to be very low (<0.5 at%) in both cases. ALD of the homoleptic tris-amidinate [Y(DPAMD)_3_] and tris-guanidinate [Y(DPDMG)_3_] show a notable difference in the GPC (0.8 Å *vs.* 1.3 Å), whereas their ALD window temperature margins and precursor evaporation temperatures (130 °C) are quite similar. Both processes yield high-quality Y_2_O_3_ thin films which were applied in capacitor stacks.^14,15^ It should be noted that comparison of parameters in different ALD processes might not be directly possible if the processes are not optimized in the same type of reactor with similar geometries, flow rates, temperature gradients and many other ALD conditions.^16^ This is directly apparent when comparing water assisted ALD processes using the heteroleptic yttrium isopropyl-cyclopentadienyl amidinate [Y(^iPr^Cp)_2_(DPAMD)] precursor which was employed in three different reactors: the growth per cycle (GPC) of 0.4–1.3 Å, ALD-Windows (175–200 °C; 200–350 °C; 350–450 °C), bubbler temperatures (120–150 °C) and composition (C,N < 0.5 at% – C ≈ 3.7 at%) vary significantly, making a thoughtful and direct comparison to other precursors nearly impossible.^17–20^ Overall, there are different precursors reported for water-assisted ALD of Y_2_O_3_ (Fig. 1), which share the same drawback of low volatilities and thus necessitates high precursor evaporation temperatures that might limit their applicability in low-temperature ALD processes.

**Scheme 1:**
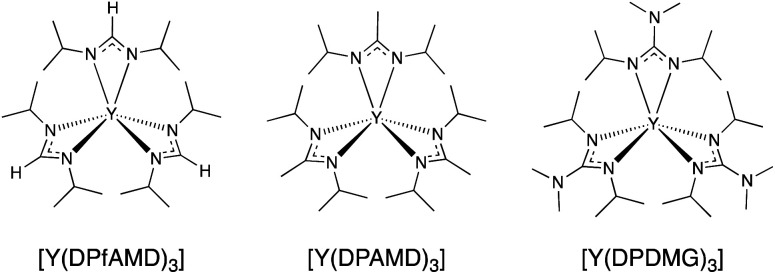
Molecular structures of the yttrium precursors [Y(DPfAMD)_3_] (this study), [Y(DPAMD)_3_], [Y(DPDMG)_3_].

Thus, it is not only necessary to identify and develop new precursors and processes for the ALD of Y_2_O_3_ films but also equally important to optimize the processes employing different precursors in the same reactor setup to check reproducibility and conveniently compare the behavior of precursors and processes. This will enhance the understanding of precursor chemistry and process characteristics suiting the targeted applications. As shown earlier in studies by Rouf *et al.*^21^ on the ALD of InN and Kim *et al.*^22^ on In_2_O_3_, the change of the endocyclic substituents on the respective amidinate and guanidinate backbones (*e.g.* –H, –Me and –NMe_2_) of the homoleptic complexes revealed a superior performance of the formamidinate derivative (–H) complexes in the corresponding processes. Herein, we report on a new water-assisted ALD process with the homoleptic precursor tris(*N*,*N*′-diisopropyl-formamidinato)yttrium(iii) [Y(DPfAMD)_3_] for the formation of Y_2_O_3_ thin films, which features distinct advantages such as higher volatility and favorable processing characteristics compared to the other known yttrium precursors of the same family. For [Y(DPfAMD)_3_], the typical ALD characteristics were verified, the resulting thin films thoroughly analyzed and finally applied in a metal–insulator–semiconductor (MIS) capacitor stack to investigate the electrical properties. Additionally, in this study, the newly developed ALD process employing [Y(DPfAMD)_3_] is directly compared to a process employing the guanidinate [Y(DPDMG)_3_] (Scheme 1) in the same reactor and under similar process conditions,^15^ which clearly underlines the superior characteristics of the formamidinate backbone for the ALD of Y_2_O_3_ thin films.

## Experimental section

The synthesis and handling of all reagents and compounds was carried out utilizing standard Schlenk protocols using Ar as an inert gas to prevent contact with ambient air and moisture. The precursors were handled and stored inside a MBraun 300 Glovebox system and the solvents were dried by a MBraun solvent purification system (SPS) and stored under inert gas atmosphere. All commercially available reagents were used without further purification. The two reported precursors [Y(DPDMG)_3_] and [Y(DPAMD)_3_] were synthesized according to literature known procedures by Milanov *et al.* and de Rouffignac *et al.*,^14,23^ while [Y(DPfAMD)_3_] is commercially available. Electronic ionization mass spectra (EI-MS) were recorded by a Varian MAT spectrometer with direct sample injection. Thermogravimetric analysis (TG) was carried out with a Seiko Exstar TG/DTA 6500SII under a nitrogen flow (300 ml min^−1^) and a heating rate of 5 K min^−1^ using approx. 10 mg of each compound. The vapor pressure of the compounds was determined using stepped isothermal TGA. This approach is based on a study by Kunte *et al.*^24^ For the depositions of Y_2_O_3_ thin films, 2′′ p-type Si(100) substrates with native oxide (SiO_*x*_, ≈2 nm) were used. The ALD experiments were carried out using a ASM Microchemistry F-120 reactor. The temperature of the precursor (200 mg for each deposition) was kept at 95 °C for depositions carried out from 100–275 °C and 98 °C for depositions at 300–325 °C with an active flow of 300 sccm N_2_. The water reservoir was always held at room temperature. Optimized pulse-purge sequence of the ALD process for the determination of the ALD window was 5 s of precursor pulse, 60 s of precursor purge, 5 s of water pulse and 30 s of water purge. The final optimized sequence at a deposition temperature of 300 °C is 5 s of precursor pulse, 10 s of precursor purge, 1 s of water pulse and 30 s of water purge. The thickness of the thin films was determined *via* spectral reflectance using a spectrometer F20 from Filmetrics. Grazing incidence X-ray diffraction (GI-XRD) was carried out using a PANalytical X'pert pro diffractometer using full 2′′ substrates. Thin film density and the critical angle was derived *via* X-ray reflectometry (XRR; Bruker D8 Discover XRD) with Cu-K_α_ radiation (1.5418 Å) in a *Θ*–2*Θ* locked coupled mode, while 2*Θ* was increased from 0.1 to 3 with a step size of 0.01. Rutherford backscattering spectrometry (RBS) analysis and nuclear reaction analysis (NRA) were performed at the RUBION, Central Unit for Ion Beams and Radionuclides at the Ruhr University Bochum. For RBS, a 2.0 MeV ^4^He^+^ ion beam with an intensity of 20–40 nA was directed to a sample with an angle of 7°. The scattered particles were detected by a solid-state detector at 160°. NRA was performed to obtain the concentration of elements with a low atomic number like C, N and O. The concentration was obtained after an induced nuclear reaction of the light elements by a 1.0 MeV deuteron beam and detection of the emitted protons at an angle of 135°. A 6 μm Ni foil was used to shield the detector from scattered deuterons. The beam penetrates the whole thin film and is stopped in the sample substrate. The software suite SIMNRA was used to determine the concentration of the elements in the thin film, by using the data obtained by the RBS and NRA measurements.^25^ X-ray photoelectron spectroscopy (XPS) was carried out in a PHI 5000 instrument. The X-ray source was operated at 10 kV and 24.6 W using Al Kα (1486.6 eV) radiation with a 45° electron take-off angle. The kinetic energy of electrons was analyzed with a spherical Leybold EA-10/100 analyzer using a pass energy of 18 eV. After measurements for the as introduced sample were completed, the surface was subjected to Ar^+^ sputtering (1 min, 2 kV (2 × 2)). The samples were analyzed by a combination of survey scans and core level scans for peaks of interest. Step widths were adjusted to 0.5 eV for each survey scan and 0.05 eV for the core level scans. All binding energies of yttrium Y 3d, oxygen O 1s and other core levels were referenced to the Fermi edge position. The analysis chamber pressure was maintained at <10^−7^ mbar. The deconvolution analysis was completed with a Shirley background processing and Gaussian functions using UniFit 2017 software. The topography of the Y_2_O_3_ films was characterized by means of atomic force microscopy (AFM, Digital Instruments, Nanoscope V). Electrical characterization was carried out on metal–insulator–semiconductor (MIS) capacitors. For this, 20 nm thick Y_2_O_3_ film was deposited at *T* = 300 °C on a p^+^-type Si(111) substrate. 70 nm thick Pt gate electrodes were e-beam evaporated onto the Y_2_O_3_ films through a shadow mask. The diameter of the gate electrodes amounts to 70 μm. The capacitance–voltage (*C*–*V*) characteristics were measured using an Agilent E42821 A LCR meter. For the current–voltage measurements of the MIS structures a semiconductor parameter analyzer (HP Agilent 4156B) was used.

## Results and discussion

### Comparative mass spectrometry studies

**Scheme 2:**
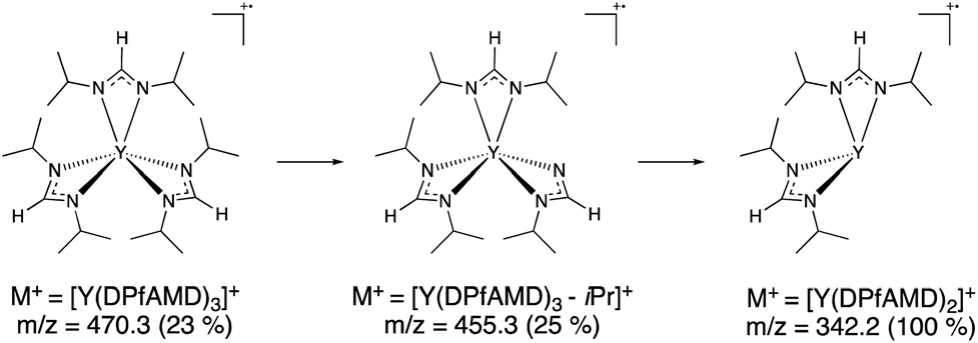
Proposed EI-MS fragmentation pattern for [Y(DPfAMD)_3_] showing the most stable fragment [Y(DPfAMD)_2_]^+^.

To gain a first insight into possible differences of the precursor chemistry between [Y(DPfAMD)_3_], [Y(DPAMD)_3_] and [Y(DPDMG)_3_], electron-impact mass spectrometry (EI-MS) was carried out (see ESI, Fig. S1†).^14,26–28^ Like its homologues, [Y(DPfAMD)_3_] is monomeric in the gas phase under these conditions indicated by the molecular ion peak M^+^ at *m*/*z* = 470.3 (rel. abund. 22%) with a consecutive fragmentation by the loss of one formamidinate ligand yielding the fragment [Y(DPfAMD)_2_]^+^ at *m*/*z* = 343.2 (base peak, 100%) (Scheme 2).

The most stable fragment [Y(DPfAMD)_2_]^+^ is then further split into smaller fragments, however the loss of another complete formamidinate ligand to [Y(DPfAMD)]^+^ at *m*/*z* = 216.0 was not observed. Also, a peak for the lone formamidinate [DPfAMD]^+^ ligand was not detected at the expected *m*/*z* = 143.2, which indicates that it directly splits into smaller and more stable fragments (Table 1). The structurally related amidinate [Y(DPAMD)_3_] exhibits a similar fragmentation behavior with an observable M^+^ peak at *m*/*z* = 512.3, a [Y(DPAMD)_2_]^+^ fragment at *m*/*z* = 371.2, a [Y(DPAMD)]^+^ fragment peak visible at *m*/*z* = 230.1 and no fragment visible at *m*/*z* = 157.2 for [DPAMD]^+^. On the contrary, the fragmentation of the yttrium guanidinate [Y(DPDMG)_3_] proceeds from the M^+^ peak at *m*/*z* = 599.4 with the loss of one ligand to [Y(DPDMG)_2_]^+^ at *m*/*z* = 429.2 and the loss of another ligand to [Y(DPDMG)]^+^ at *m*/*z* = 259.0.

**Table 1:** Selected fragments with their respective *m*/*z* ratios and relative abundance for [Y(DPfAMD)_3_], [Y(DPAMD)_3_] and [Y(DPDMG)_3_]

Compound	*m*/*z* (rel. abund.)		
	M^+^ = [ML_3_]^+^	[ML_2_]^+^	[ML]^+^
[Y(DPfAMD)_3_]	470.3 (23%)	343.2 (100%)	Not observed
[Y(DPAMD)_3_]	512.3 (15%)	371.2 (100%)	157.2 (6%)
[Y(DPDMG)_3_]	599.4 (31%)	429.2 (85.9%)	259.0 (29%)

Additionally, a peak assignable to the ligand [DPDMG]^+^ was found at *m*/*z* = 171.1 in combination with numerous smaller fragments observed at *m*/*z* <170 compared to [Y(DPAMD)_3_] and [Y(DPDMG)_3_]. The different fragmentation patterns of the respective yttrium precursors suggest that [Y(DPfAMD)_3_] and [Y(DPAMD)_3_] behave similar under EI-MS conditions with the most abundant fragment [M(L)_2_]^+^ and a lower number of small fragments compared to the fragmentation pattern of [Y(DPDMG)_3_] which additionally features the [M(L)]^+^ fragment and a higher number of fragments at lower *m*/*z*. Possible rearrangements such as carbodiimide deinsertion (CDI^+^ at *m*/*z* = 126.2 for [Y(DPDMG)_3_]) are potentially blocked by more stable C–H and C–CH_3_ bonds compared to the C–NMe_2_ bond within the N–C–N backbone,^29^ which might explain a higher overall stability of [Y(DPfAMD)_3_], [Y(DPAMD)_3_] and their related fragments compared to [Y(DPDMG)_3_].

### Comparative thermogravimetric studies

**Fig. 2:**
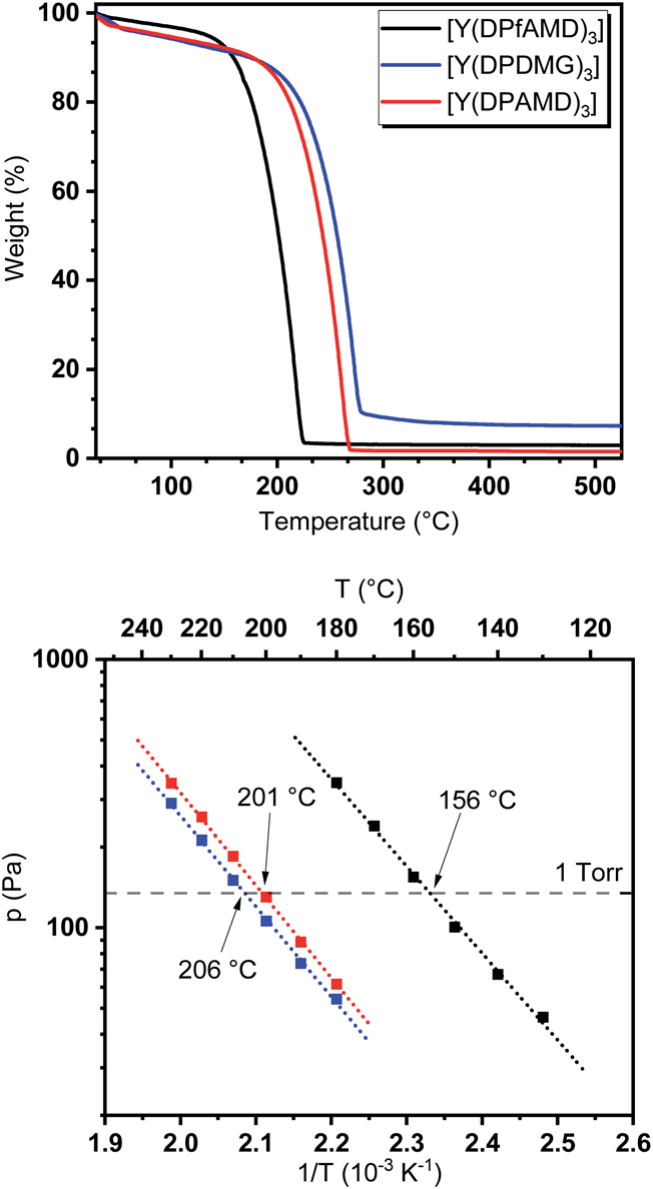
Top: TG analysis of [Y(DPfAMD)_3_] (black), [Y(DPDMG)_3_] (blue) and [Y(DPAMD)_3_] (red). Bottom: vapor pressure measurements for all three complexes. The dotted grey line represents the 1 torr vapor pressure.

To evaluate the behavior of the precursors under thermal exposure, thermogravimetric (TG) measurements were performed (Fig. 2). While all complexes feature a single-step evaporation, [Y(DPfAMD)_3_] shows an onset of volatilization at 151 °C (derived *via* tangents) which is significantly lower than [Y(DPAMD)_3_] at 197 °C and [Y(DPDMG)_3_] at 209 °C, respectively.

The higher volatility for [Y(DPfAMD)_3_] is accompanied by a low residual mass (3%) which indicates high thermal stability within the temperature range of evaporation. As expected, the amidinate [Y(DPAMD)_3_] has a similar residual weight of 2%, while the guanidinate [Y(DPDMG)_3_] exhibits a value of 8%. In terms of stability, a trend similar to the findings in the EI-MS studies can be derived for the compound family: the thermal stability at elevated temperatures might be higher for [Y(DPfAMD)_3_] and [Y(DPAMD)_3_] due to stronger C–H and C–C bonds within the ligand backbone compared to the C–N backbone bonds in [Y(DPDMG)_3_]. The higher volatility of [Y(DPfAMD)_3_] is further confirmed by vapor pressure curves derived from stepped isothermal TG yielding a 1 torr vapor pressure at 156 °C, while [Y(DPAMD)_3_] and [Y(DPDMG)_3_] feature a significantly higher temperature of 201 °C and 206 °C, respectively. The lower 1 torr vapor pressure temperature for [Y(DPfAMD)_3_] might be caused by a smaller contribution of van der Waals interactions in the solid state during evaporation due to the small –H substituent in the N–C–N backbone and a lower molecular weight.^30^ The superior thermal properties namely, enhanced thermal stability, high volatility and intrinsic reactivity render the yttrium formamidinate [Y(DPfAMD)_3_] as an interesting and promising alternative precursor to the already established [Y(DPAMD)_3_] and [Y(DPDMG)_3_] in ALD processes.

### Y_2_O_3_ ALD process development with [Y(DPfAMD)_3_] and water

**Fig. 3:**
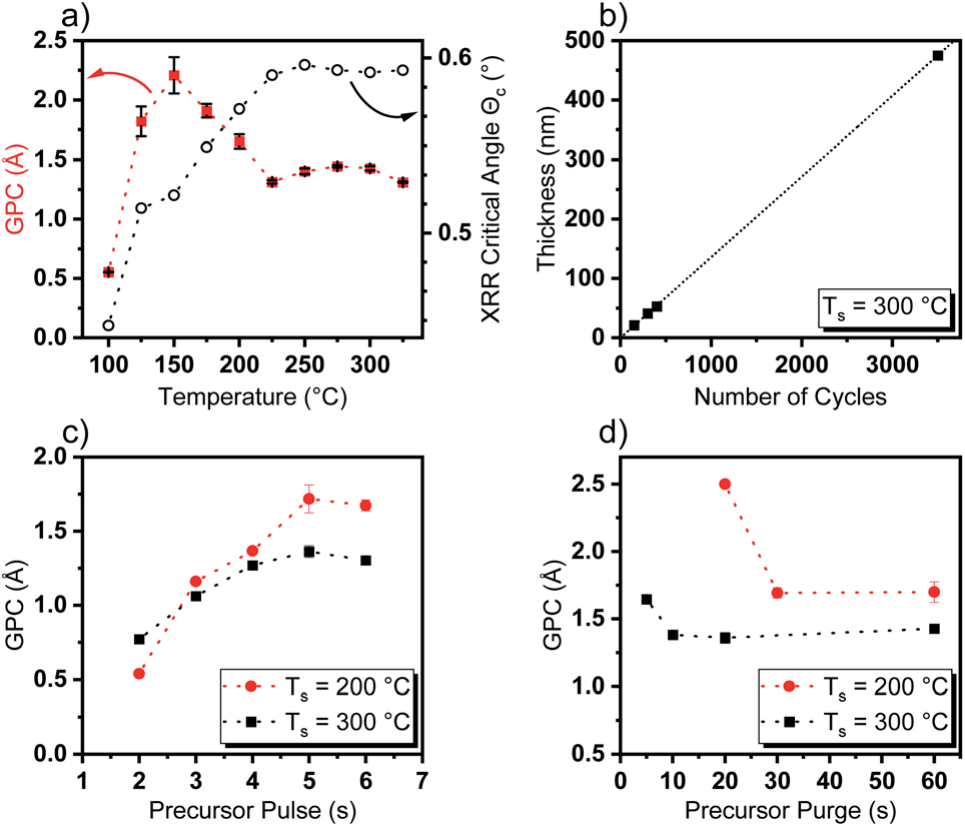
Dependence of (a) GPC on temperature and the critical angle *Θ*_c_ obtained from XRR and (b) thin film thickness as a function of number of applied cycles including a linear fit (*R*^2^ = 0.9999). (c) Dependence of GPC *versus* precursor pulse length at 200 °C (red) and 300 °C (black). (d) Dependence of GPC *versus* precursor purge length at 200 °C (red) and 300 °C.

To evaluate the performance of [Y(DPfAMD)_3_] in a thermal water-assisted ALD process for the formation of Y_2_O_3_ thin films, a thoughtful process development had to be carried out to find optimal process parameters like ALD window and surface saturation characteristics. A saturation behavior of [Y(DPfAMD)_3_] on 2′′ Si(100) substrates was reached after 5 s of precursor pulse and 10 s precursor purge at a substrate temperature of *T*_s_ = 300 °C with a GPC of 1.35 Å (as shown in Fig. 3c and d). For these parameters, the film thickness linearly increases with a slope of 0.136 nm per cycle according to a linear fit (*R*^2^ = 0.9999) (Fig. 3b). Interestingly, a comparable saturation behavior is achieved for [Y(DPDMG)_3_] which saturates after 4 s of precursor exposure at *T*_s_ = 225 °C and a GPC of 1.10 Å but with a substantially higher precursor evaporation temperature of *T*_p_ = 130 °C which is the same for [Y(DPAMD)_3_]. The lower precursor evaporation temperature of *T*_p_ = 95 °C for [Y(DPfAMD)_3_] to reach saturation of the surface, underlines the higher volatility of this precursor in agreement with the TGA experiments. It is interesting to note that, [Y(DPfAMD)_3_] can be employed at higher substrate temperatures up to 325 °C, whereas [Y(DPDMG)_3_] and [Y(DPAMD)_3_] presumably started to decompose above 275 °C, which led to higher GPCs and higher impurity levels.^14,15^ The GPC remains nearly constant at 1.35 Å from 225 °C to 325 °C, while below 225 °C an increase in the GPC to 1.7 Å and a decrease in film homogeneity from 225 °C to 200 °C is apparent (Fig. 3a). Even though an increase in the GPC at lower temperatures normally can be attributed to condensation of the precursor, the saturation of the precursor at 200 °C is also observed after a 5 s precursor pulse, but a higher precursor purge time (30 s minimum) is needed for obtaining saturation without notable physisorption component (Fig. 3d). At a substrate temperature of 300 °C, a precursor purge of 10 s is sufficient to remove excess precursor and by-products which might indicate faster surface kinetics at higher temperatures.

Additionally, this might be explained by a shift from chemisorption to physisorption of the precursor on the surface below 225 °C, which would explain the higher GPC and yet saturation behavior of the precursor.^31^ The GPC steadily increases to 2.3 Å below 200 °C and below 150 °C the GPC drops considerably and reaches a minimum value of 0.51 Å at 100 °C where the temperature is not effectual to reach sufficient adsorption of the precursor on the substrate surface. To obtain an initial insight into the properties of the Y_2_O_3_ films deposited within the ALD window, X-ray reflectivity (XRR) curves were recorded and the critical angle *Θ*_c_ was extracted as a measure for the thin film density. An increase of *Θ*_c_ with increasing temperature from *Θ*_c_ = 0.45° at 100 °C to *Θ*_c_ = 0.59° at 225 °C could be observed which remains constant at *Θ*_c_ = 0.59 up to a deposition temperature of 325 °C (Fig. 3a). This further supports the proposition of an ALD window with dense Y_2_O_3_ films reaching from 225 °C to 325 °C, whereas below this temperature range the measured critical XRR angle indicates a lower density. This, besides other factors, might be caused by a higher level of impurities and increased O/Y stoichiometry of the films which indicates significant hydroxyl (–OH) incorporation as shown later in the section “Compositional analysis”. Overall, ALD with [Y(DPfAMD)_3_] enables the usage of lower precursor evaporation temperatures and higher deposition temperatures when compared to processes with [Y(DPDMG)_3_] and [Y(DPAMD)_3_].

### Thin film characterization

To thoroughly identify the quality and properties of the deposited Y_2_O_3_ thin films and its properties using [Y(DPfAMD)_3_] in terms of crystallinity, morphology and composition, complementary analyses were carried out and the results are subsequently discussed. If not stated otherwise, the thin films were deposited at 300 °C with the optimized parameters discussed before.

#### Thin film crystallinity

**Fig. 4:**
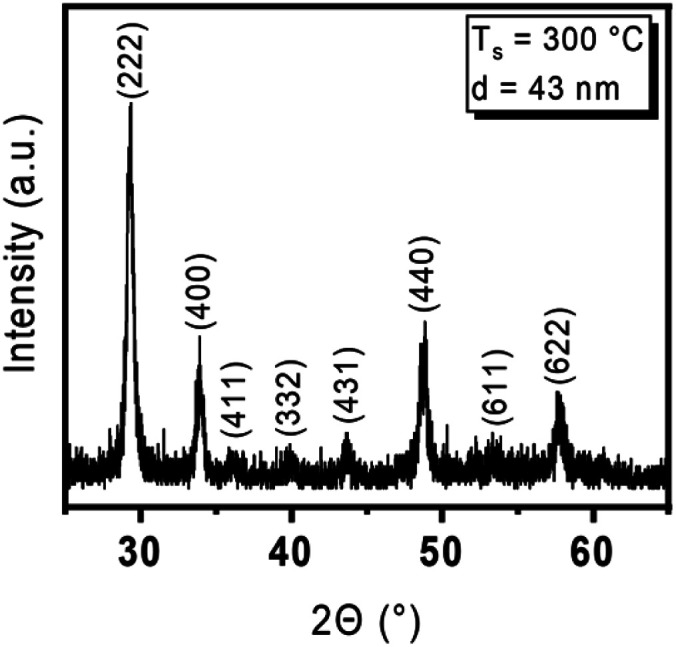
GI-XRD pattern from a 43 nm Y_2_O_3_ thin film deposited at 300 °C on Si(100) and reflections assigned according to ICSD 185295.

To gather information on the crystallinity of the Y_2_O_3_ thin films deposited at 300 °C, grazing-incidence X-ray diffraction (GI-XRD) was carried out. The analysis of a 43 nm Y_2_O_3_ film on Si(100) revealed polycrystallinity with face-centred cubic packing (fcc) whereby the (222), (400), and (622) reflections being strongly pronounced (Fig. 4).

#### Thin film density and morphology

**Fig. 5:**
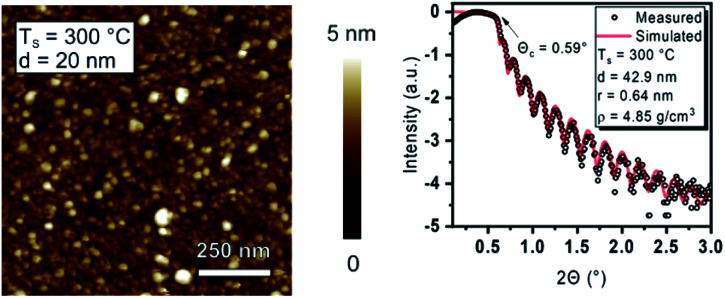
Left: AFM image of a 20 nm Y_2_O_3_ thin film deposited at 300 °C on Si(100). Right: XRR curve from the latter thin film with the simulated curve and obtained thin film parameters such as thickness, roughness, and density.

XRR measurements on a 43 nm Y_2_O_3_ film deposited at 300 °C delivered typical Kiessig fringes and a critical angle at *Θ*_c_ = 0.59° (Fig. 5, right),^32^ which correlates to a Y_2_O_3_ film density of 4.85 g cm^−3^. The derived density is close to the crystalline bulk density of Y_2_O_3_ (5.03 g cm^−3^),^33^ and the deviation might be caused by crystal grains from the pronounced polycrystalline nature of the thin film, while additionally hydroxyl (–OH) incorporation as an intrinsic feature of the water-assisted ALD process could play a role here as shown later by XPS analysis.^34^ Moreover, the obtained density of 4.70 g cm^−3^ for films deposited at 200 °C is still considerably higher than those obtained for the ALD process with [Y(DPDMG)_3_] (4.24 g cm^−3^) at 200 °C. From the slope of the XRR fringes a low roughness of *r* = 0.64 nm is derived which is typical for ALD growth and close to the roughness of the uncoated Si(100) substrate with *r* = 0.3 nm.

To confirm the low roughness and assess the overall morphology of Y_2_O_3_ films (thickness of 20 nm) grown at 300 °C, AFM measurements were conducted (Fig. 5, left). A smooth surface with an RMS roughness of *r* = 0.63 nm (1 μm *×* 1 μm) is observed. The Y_2_O_3_ films (20 nm) obtained by using [Y(DPDMG)_3_] at *T*_s_ = 200 °C feature a slightly lower but not significantly deviating RMS roughness of *r* = 0.55 nm, which might be explained by a lower degree of polycrystallinity as usually observed at lower *T*_s_ in ALD. Interestingly, AFM measurements on 20 nm Y_2_O_3_ films grown at 200 °C revealed a RMS roughness of *r* = 0.60 nm that is only slightly higher than the comparable process, but lower than the value obtained at 300 °C. Although the morphological and crystalline nature of the Y_2_O_3_ confirm the characteristics typically seen for ALD processes, a thoughtful compositional analysis is still necessary and is discussed in the following section.

### Compositional analysis

#### RBS and NRA

The purity in terms of thin film stoichiometry and contamination levels on the atomic scale are accessible using RBS and NRA (Table S1†). Accordingly, for Y_2_O_3_ films deposited at 300 °C, compositional analysis *via* RBS revealed a O/Y ratio of 1.7, with 63 at% oxygen and 37 at% yttrium, while impurities such as nitrogen and carbon were near the detectable limits of NRA with <1 at%. The O/Y ratio of 1.7 is slightly higher than the expected ratio of 1.5 for Y_2_O_3_ at this temperature, which might be explained by hydroxyl (–OH) incorporation as proposed previously for our water-assisted ALD process with [Y(DPDMG)_3_] (O/Y = 2.0 at *T*_s_ = 225 °C).^15^ This was the case for films deposited with [Y(DPAMD)_3_] as reported by Rouffignac *et al.*, where an O/Y stoichiometry of 2.0 for the as-deposited films at 270 °C and a stoichiometry of 1.7 was found after *in situ* capping with Al_2_O_3_ to prevent the adsorption of hydroxyls from the ambient atmosphere.^14^ Although the O/Y ratio remains constant at 2.0 from 200 °C to 250 °C for our new process with [Y(DPfAMD)_3_], it lowers to 1.6 at 325 °C and rises to 2.3 at 150 °C. This furthermore indicates that the deposition temperature plays a crucial role for the stoichiometry of the Y_2_O_3_ films and suggests, that a higher temperature might be more effective in removing the excess of adsorbed –OH species from the near surface of the film during deposition. Depositing thicker layers (470 nm) of Y_2_O_3_ at 300 °C leads to an ideal value of 1.5. It must be considered that the longer exposure to higher temperatures during longer depositions due to the increased cycle amount might induce annealing effects and can consequently be responsible for the observed O/Y ratio. Furthermore, Y_2_O_3_ is known to alter its surface upon exposure to the ambient with moisture and undergo additional hydroxylation which is more prevalent and causes an increased O/Y value for thinner films as investigated by RBS where the whole depth of the sample is penetrated by the beam.^35^ From these results it is apparent that a ratio closer to that of the bulk of Y_2_O_3_ could be achieved using [Y(DPfAMD)_3_] which was not the case for as-deposited films using either [Y(DPDMG)_3_] or [Y(DPAMD)_3_] at lower temperatures. Moreover, the new process employing [Y(DPfAMD)_3_] reduced the contamination levels with C and N below the detectable limits of NRA, which was not achieved to the same extent with [Y(DPDMG)_3_] (C: 2–5 at% and N: 2–3 at% at *T*_s_ = 225 °C).

#### XPS

The nature of chemical species within Y_2_O_3_ thin films deposited with optimized process parameters at 300 °C for a representative 40 nm thick film was investigated by XPS analysis. Hereby, the sample was exposed to the ambient as little as possible to prevent alteration of its surface through interactions with moisture and carbon dioxide which has been found to cause significant surface hydroxylation as discussed earlier.^36,37^

**Fig. 6:**
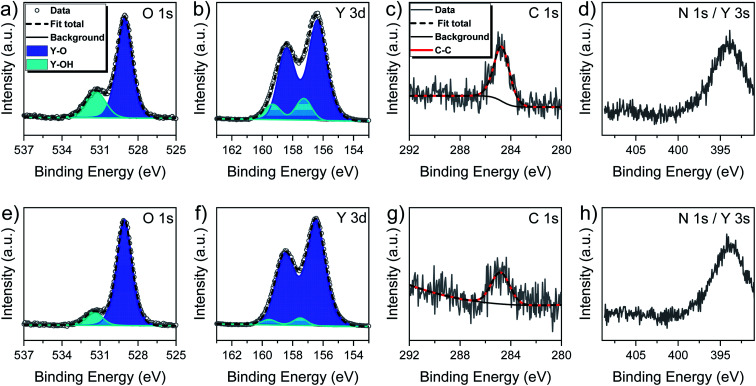
XPS core levels recorded for a 60 nm thick Y_2_O_3_ thin film grown at 300 °C (upper row: as deposited; lower row: after sputtering). (a + e) O 1s core level regions, (b + f) Y 3d core level regions, (c + g) C 1s core level regions and (d + h) overlapping N 1s and Y 3s core level regions.

The survey spectra recorded for the as introduced and sputtered surfaces (ESI, Fig. S3†) revealed the presence of all signals expected for yttrium and oxygen. While a weak signal originating from adventitious carbon was seen for the as introduced surface, nitrogen related signals were not found. The composition of the film prior to sputter treatment and thereafter is given in Table 2, while Fig. 6 contains scans of the O 1s, Y 3d, C 1s and Y 3s core level regions.

**Table 2:** Overview of the compositional values for the elements C, N, O and Y determined by XPS for a 40 nm Y_2_O_3_ thin film grown at 300 °C on Si(100)^a^

		C (at%)	N (at%)	O (at%)	Y (at%)	Si (at%)	O/Y
40 nm Y_2_O_3_ on Si(100)	As introduced	4.8	n.d.	58.4	36.8	n.d.	1.59
	Sputtered	2.5	n.d.	52.7	44.8	n.d.	1.18

a n.d.: not detected.

Owing to the short exposure time to the ambient, the amount of adventitious carbon on the untreated surface was found to be rather low with around 4.8 at%. Oxygen was found to be predominant with 58.4 at% followed by yttrium with 36.8 at%. This resulted in a O/Y ratio of 1.59 which is well within the expected range, supporting the assumption that the water assisted ALD process leaves a partially hydroxylated surface after the process and is moreover in accordance with the results obtained by NRA. After sputtering the carbon contamination level decreased to 2.5 at% while the O/Y ratio drops down to 1.18 as a consequence of preferential oxygen sputtering.^38^

The oxygen core level regions presented in Fig. 6a for the as deposited and in Fig. 6e for the sputtered surface comprises of signals with two well distinguishable components of which the ones with a lower binding energy of 529.1 eV each are by far predominant and assigned to Y–O lattice oxygen.^15,39^ For the as deposited surface, the second component is found at a higher binding energy of 531.3 eV and resembles Y–OH hydroxyls,^36,39^ while this contribution is reduced and slightly shifted to 531.5 eV after sputtering. Thus, the presence of a minor amount of Y–OH species on the surface and in the bulk is confirmed and can expectedly be considered as an intrinsic feature of the ALD process. More precisely, the share of the Y–OH component for the as-deposited surface on the total integral of the O 1s core level amounted 24.5% and was reduced to 11.6% after sputtering. In first approximation, the low contribution of hydroxyls is concomitant with rather low hydrogen inclusion in the film, but a precise determination of the hydrogen content is not possible by XPS. Interestingly, the share of the Y–OH component in the overall integral in the bulk is significantly lower for this new water assisted ALD process with [Y(DPfAMD)_3_] than for our prior process with [Y(DPDMG)_3_]. We priorly proposed that it was the steric demand of the guanidinate backbone that was responsible of the high content of Y–OH in the film as it could shield some hydroxyl groups during the process so that they could not react with incoming precursor molecules.^15^ Moreover, for the water assisted ALD process with [Y(DPAMD)_3_] it was reported that directly after deposition at 275 °C and capping with Al_2_O_3_ to prevent further incorporation of –OH species from air, a significant amount of hydroxyl species are remaining in the bulk of the films.^14^ The formamidinate skeleton on the contrary has been demonstrated to be less sterically demanding and more flexible in the ALD of In_2_O_3_ thin films by the closely related precursors [In(DPfAMD)_3_] and could thus facilitate inclusion of less Y–OH in the film, which could be confirmed in the parent study.^22^ Congruously, less inclusion of hydrogen in the Y_2_O_3_ films is enabled by utilization of [Y(DPfAMD)_3_] compared to its congeners.

The Y 3d core level spectra of the as deposited (Fig. 6b) and sputtered (Fig. 6f) film surface did not contrast the insights gained from the O 1s core region. In both cases the Y 3d region showed a signal feature with well resolved spin–orbital components. The signals themselves comprised two contributions described as doublets. In accordance with reported literature,^15,40^ fitting was performed with a fixed energy separation of 2.05 eV between 3d_5/2_ and 3d_3/2_ components and intensity ratios between 3d_3/2_ and 3d_5/2_ of about 0.7. Binding energies for the Y 3d_5/2_ component of Y–O are typically found in a range from 156.4–156.8 eV while the Y 3d_5/2_ component of Y–OH species have been reported to be found between 157.3–158.0 eV.^28,41,42^

In this study the Y 3d_5/2_ component for Y–O is located at 156.4 eV and the one for Y–OH species at 157.3 eV for the as deposited surface. Expectedly, latter species only possesses a minor share in the overall integral and it is observed that this share is decreased even more after sputtering while the positions remain roughly the same with 156.5 eV and 157.6 eV respectively. The C 1s core level spectra before and after sputtering (Fig. 6c and g) solely contain one species at 284.8 eV that originates from C–H type impurities. Contributions from carbonates were not seen and consequently not considered to be a factor in either the O 1s or Y 3d signal fitting. Lastly, the overlapping N 1s/Y3d core level regions (Fig. 6d and h) did not provide evidence for the presence of nitrogen in the film, which might have been observable as a shoulder component in the Y 3s signal. Thus, the findings from XPS are in overall good alignment with prior RBS/NRA investigations and evince that pure Y_2_O_3_ thin films can be deposited in a wide temperature range using [Y(DPfAMD)_3_] and water, while nearly stoichiometric films can be obtained at a deposition temperature of 300 °C without further post-treatment such as annealing or capping of the films.

### Functional properties

#### Optical characterization

**Fig. 7:**
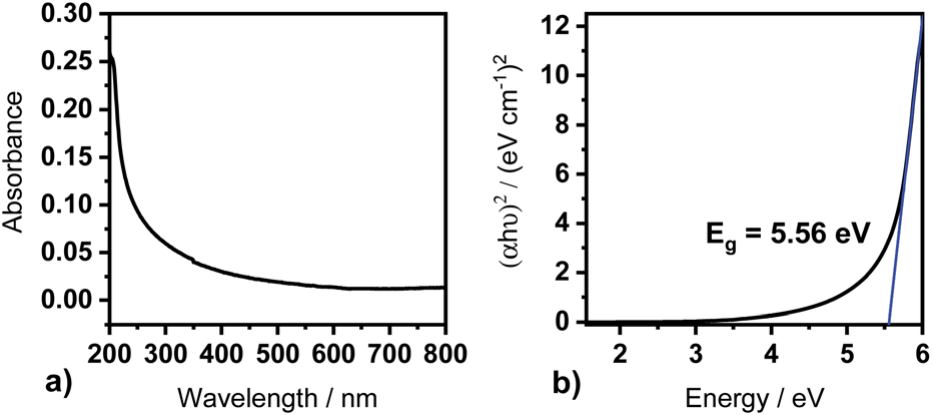
UV-Vis measurements conducted with Y_2_O_3_ films (20 nm) deposited at 300 °C. (a) Absorbance in dependence of the wavelength and (b) Tauc plot for the determination of the optical direct band gap from the (*αhν*)^2^ term.

As Y_2_O_3_ finds its application in optoelectronic devices and is used as a high-*κ* dielectric material in MOSFETs, an analysis and estimation of the allowed direct band gap energy (*E*_g_) is of high interest. A 20 nm Y_2_O_3_ film was deposited at *T*_s_ = 300 °C on fused silica substrates and was subjected to UV/Vis measurements (Fig. 7). The Y_2_O_3_ thin film presents a low absorption of <10% from wavelengths of 800 nm to 300 nm, after which the absorption increases to <40% at 200 nm (Fig. 7a).

The Tauc plot represents an option to obtain the optical band gap energy for allowed direct band gaps from the (*αhν*)^2^ term in dependence of the energy of the corresponding transmitting light beam (Fig. 7b). An extrapolation of the linear regime of the curve to the *X*-axis gives the direct optical band gap energy. For this thin Y_2_O_3_ film we derived a direct optical band gap energy of *E*_g_ = 5.56 eV, which is not only in accordance with band gaps derived from films of processes with [Y(DPDMG)_3_] (*T*_s_ = 225 °C, 20 nm), but also with literature reported optical band gaps (*E*_g_ = 5.5–5.8 eV).^1^

#### Electrical characterization

To demonstrate that the Y_2_O_3_ thin films grown at 300 °C have the potential to be utilized in microelectronic devices, the *C*–*V* and *I*–*V* characteristics were examined in the form of a metal–insulator–semiconductor (MIS) structure with a Pt top electrode.

**Fig. 8:**
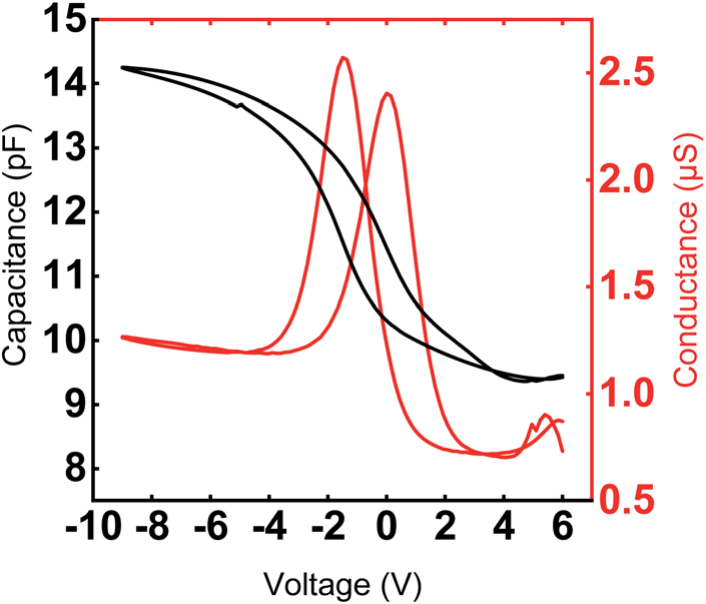
Typical *C*–*V* and *G*–*V* characteristic of Y_2_O_3_ MIS capacitors with a diameter of 70 μm measured at 1 MHz. The 24 nm Y_2_O_3_ film was deposited at 300 °C.

Typical *C*–*V* and *G*–*V* characteristics (*f* = 1 MHz) of a Y_2_O_3_ MIS device is depicted in Fig. 8. The permittivity is derived from the maximum capacitance in the accumulation regime at negative bias voltages. Considering a native 2 nm layer of SiO_*x*_ on top of the p^+^-Si substrate, we estimate a permittivity of 13.9 with a standard deviation of *σ* = 0.98. The measured value corresponds to the oxide capacitance since the series capacitance of the depletion zone is negligible. The value of the permittivity of the Y_2_O_3_ film is clearly enhanced compared to the results of Niinistö *et al.* (*k* = 10),^13^ Rouffignac *et al.* (*k* = 11–12) with [Y(DPAMD)_3_], our group (*k* = 11) with [Y(DPDMG)_3_] and is in line with data reported by Lee *et al.* (*k* = 14).^14,15,17^

**Fig. 9:**
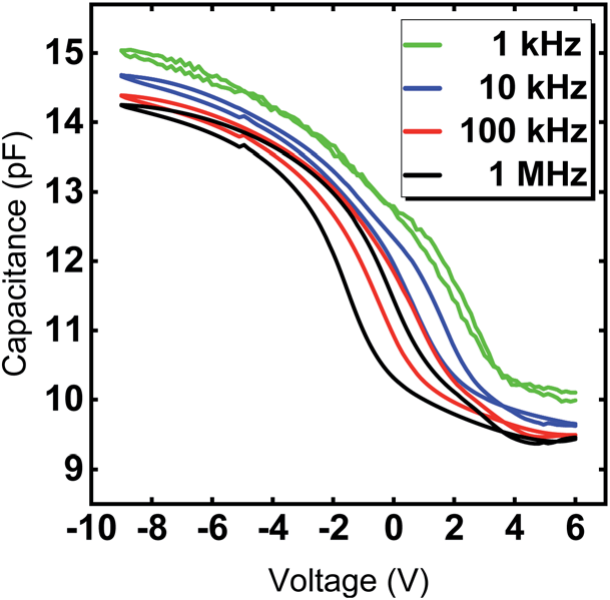
Capacitance–voltage curves of Y_2_O_3_ MIS capacitors with a diameter of 70 μm for frequencies *f* = 1 kHz, 10 kHz, 100 kHz and 1 MHz. The 24 nm Y_2_O_3_ film was deposited at 300 °C.

The hysteretic behavior in a *C*–*V* curve of a MIS device arises due to the presence of mobile charges and charge injection into the gate oxide from the semiconductor. Mobile charges would give rise to a hysteresis in the clockwise direction and the charge injection case would give rise to a counterclockwise hysteresis for a sweep from positive to negative gate voltage in a p-type substrate. Fig. 8 clearly shows the shift of the flat band voltage towards more positive gate voltages during the reverse sweep from negative to positive gate bias exhibiting a clockwise hysteretic behavior for the Y_2_O_3_ layers. Such a shift in the *C*–*V* curve could plausibly arise from the presence of negative charges accumulating at the interface.^43^ In our case, the presence of –OH species accumulated at the interfacial layer could contribute to the hysteretic behavior and is consistent with the RBS and XPS results, which indicate a higher oxygen content compared to the ideal stoichiometry of Y_2_O_3_. The *V*_FB_ derived for several devices from the 1/*C*^2^*vs. V* plot was found to be in the range of −2.93 V ≤ *V*_FB_ ≤ −2.46 V at *f* = 1 MHz and indicate the non-negligible amount of negative fixed charges within the film. It is known that such instabilities in flat band voltage can be removed by subjecting the layers to forming gas treatment, which could be a possible route for further improvement of the electrical characteristics. The frequency dependent *C*–*V* characteristic of a typical capacitor device is depicted in Fig. 9, where the frequency was reduced starting from *f* = 1 MHz to *f* = 1 kHz. With decreasing measurement frequency, the capacitance slightly increases, and the flat band voltage shifts from *V*_FB_ (*f* = 1 MHz) = −5.48 V to *V*_FB_ (*f* = 1 kHz) = −2.83 V. The reduced capacitance at higher frequencies can be attributed to interface states, which cannot follow a high-frequency field and only contribute to the overall capacitance at lower frequencies. Flat-band voltage-shift and a reduced hysteresis for consecutive measurements are observed. Garvatin *et al.* ascribe such a behaviour in HfO_2_ MIS devices to deep traps for electrons.^44^ Due to discharging of the deep traps the flat band voltage shifts to more positive values. In our case we can attribute the flat-band voltage shift to the trapping of mobile charge carriers from Si substrate resulting in a band bending of the dielectric layer. These charged trap states cannot be discharged in the subsequent *C*–*V* sweeps leading to a clearly reduced hysteresis and a more positive flat band voltage.

The interface trap density *D*_it_ can be extracted using the *C*–*V* characteristic. A more sensitive approach is the conductance method proposed by Nicollian and Goetzberger.^45^ The simplified equivalent circuit in Fig. S5† of a MIS capacitor consists of an oxide capacitance (*C*_ox_), a substrate capacitance (*C*_S_), an interface trap capacitance (*C*_it_) and resistance (*R*_it_). The parallel branch in Fig. S5a† can be converted into a frequency dependent capacitance *C*_p_ in parallel with a frequency conductance *G*_p_ (Fig. S5b†), where *C*_P_ and *G*_P_ are given by eqn (1) (in ESI, S6†). *C*_it_ denotes the interface trap capacitance *C*_it_ = *q*^2^*D*_it_, *ω* = 2π*f* the angular frequency and *τ*_it_ = *R*_it_*C*_it_ the interface-trap lifetime. The interface trap density *D*_it_ is obtained by using the relation in terms of the maximum conductance *G*_p,max_ in eqn (2) (in ESI, S6†). Fig. 8 shows the measured conductance at 1 MHz for an Y_2_O_3_ MIS device. From the maximum conductance the interface trap density the *D*_it_ was extracted to be 1.25 ×10^11^ cm^−2^ (eqn (3), ESI†). It should be noted that it is rare to find values of the interface trap densities of ALD deposited Y_2_O_3_ films. For comparison we came across an interface trap density of 1.3 × 10^12^ cm^−1^ eV^−1^ determined by Lee *et al.*^17^ It is one order magnitude higher compared to our value and exhibits superior quality of the interfaces in the present work. Finally, current density as a function of the electric field (*J*–*E*) was measured for several devices to determine the leakage current and the dielectric breakdown (Fig. S4†). All devices show a high breakdown field between 4.2 and 6.1 MV cm^−1^, which satisfies the requirements of CMOS gate oxide requirements. The values comply with breakdown fields determined for ALD grown Y_2_O_3_ based on other precursors.^13,14^ The leakage current density is around 10^−7^ A cm^−2^ at 2 MV cm^−1^ (Fig. S4†) and thus, in accordance with the lower leakage currents found in processes with [Y(DPDMG)_3_].

## Conclusions

In summary, we report a new and promising water-assisted Y_2_O_3_ ALD process employing the precursor [Y(DPfAMD)_3_] that features distinct advantages over its analogous [Y(DPAMD)_3_] and [Y(DPDMG)_3_] complexes. A low 1 torr vapor pressure temperature of 156 °C combined with a high thermal stability compared to the other precursors render [Y(DPfAMD)_3_] exceptionally useful for water assisted ALD processes for the formation of Y_2_O_3_ thin films applying low precursor evaporation temperatures. The new process gives rise to a broad ALD window ranging from 225 °C to 300 °C, while the obtained films are polycrystalline, smooth and of very good compositional quality at a substrate temperature of 300 °C. The origin of the enhanced ALD window and improved compositional quality especially in terms of thin film stoichiometry of the new process compared to the already reported processes with [Y(DPDMG)_3_] and [Y(DPAMD)_3_] should be further studied with *in situ* diagnostics and theoretical studies, while the precursor [Y(DPfAMD)_3_] with the corresponding new process on its own broadens the applicability of Y_2_O_3_ ALD. The high permittivity of Y_2_O_3_ films deposited by [Y(DPfAMD)_3_] obtained from an application in MIS capacitors is one of the highest reported in literature for ALD grown Y_2_O_3_ films and the low interface trap density of 1.25 × 10^11^ cm^−2^ and low leakage current around 10^−7^ A cm^−2^ at 2 MV cm^−1^ underlines the high interface quality of the films obtained from the new ALD process developed in this study. These experiments set an exciting starting point for further in-depth comparative studies on how the ligand sphere of the employed precursor can drastically alter its physico-chemical properties which in turn influences the ALD process parameters and thus the applicability of the process. This will further enhance the understanding and relation between precursor chemistry and ALD process performance which can be applied to other material systems.

## Conflicts of interest

There are no conflicts to declare.

## Funding sources

The authors gratefully acknowledge the financial support from the Federal Ministry of Education and Research of Germany (BMBF) within the ForMikro project FlexTMDSense (16ES1096K) and by the German Research Foundation; DFG-SPP1796 (DE-790-17-1 and BO-2495/3-1) and the SFB-TR-87-B04.

## Supplementary Material

RA-011-D0RA09876K-s001
