# Supplementary material for: Atomic layer deposition of dielectric Y2O3 thin films from a homoleptic yttrium formamidinate precursor and water
Source: RSC Adv. 2021 Jan 12;11(5):2565–74. doi: 10.1039/d0ra09876k (PMC8693876; doi:10.1039/d0ra09876k)
Supplement: RA-011-D0RA09876K-s001 [file RA-011-D0RA09876K-s001.pdf]

Electronic Supplementary Material (ESI) for RSC Advances.
This journal is © The Royal Society of Chemistry 2020

Electronic Supplementary Information

Atomic layer deposition of dielectric Y_2O_3 thin films from a homoleptic yttrium formamidinate precursor and water

Nils Boysen[a], David Zanders[a], Thomas Berning[b], Sebastian M. J. Beer[a], Detlef Rogalla[c], Claudia Bock[b], Anjana Devi[a*]

[a] Inorganic Materials Chemistry, Ruhr University Bochum, 44801 Bochum, Germany (anjana.devi@rub.de)

[b] Microsystems Technology, Ruhr University Bochum, 44801 Bochum, Germany

[c] RUBION, Ruhr University Bochum, 44801 Bochum, Germany

S1. EI-MS Spectra

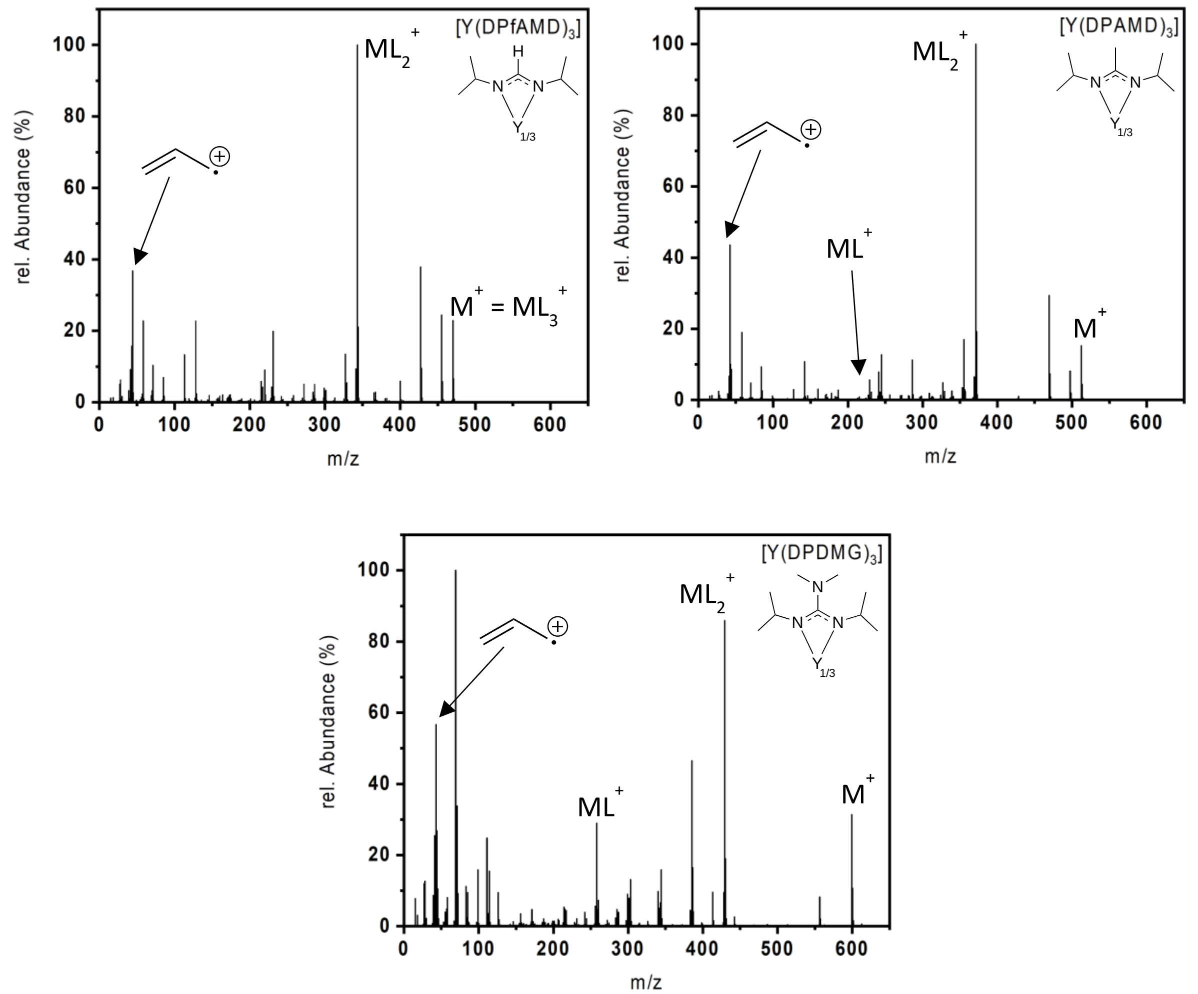

Figure S 1. EI-MS spectra with the fragmentation behavior for all three precursor molecules. The molecular peak (M^+) is the peak with the highest observable m/z ratio in all spectra.[1–4]

S3. ALD Saturation Studies

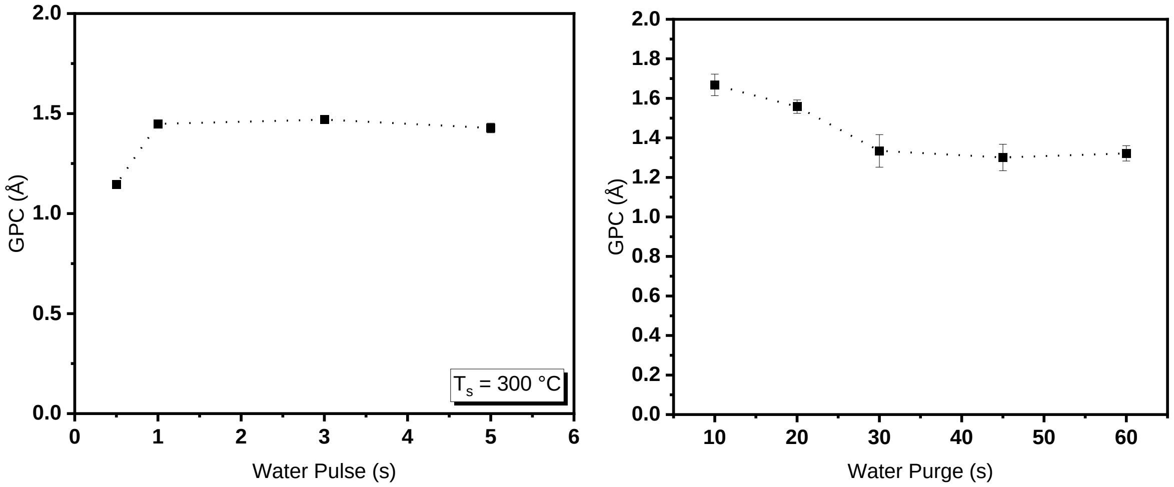

Figure S 2. Water saturation curves: Dependence of the GPC to water pulse length (left) and water purge length (right) for films deposited at 300 °C on Si(100).

S4. Composition analysis through RBS/NRA

Table S1. Overview of the atomic percentage values for the light elements (C, N and O) obtained by NRA and for yttrium obtained by RBS at different substrate temperatures T_s resulting in different oxygen to yttrium ratios.

T_s (°C)	C (at.%)	N (at.%)	O (at.%)	Y (at.%)	O/Y
**100**	19	1	71	9	7.7
**125**	6	0	69	24	2.8
**150**	5	1	66	28	2.3
**175**	4	0	67	28	2.4
**200**	2	0	65	33	2.0
**225**	1	2	64	33	2.0
**250**	1	0	65	33	2.0
**275**	< 1	1	64	35	1.8
**300**	< 1	< 1	63	37	1.7
**300**[(a)]	< 1	< 1	59	40	1.5
**325**	< 1	2	61	37	1.6

. [(a)] Y_2O_3 film thickness d = 470 nm using the same conditions as mentioned before.

S5. X-ray Photoelectron Spectroscopy (XPS)

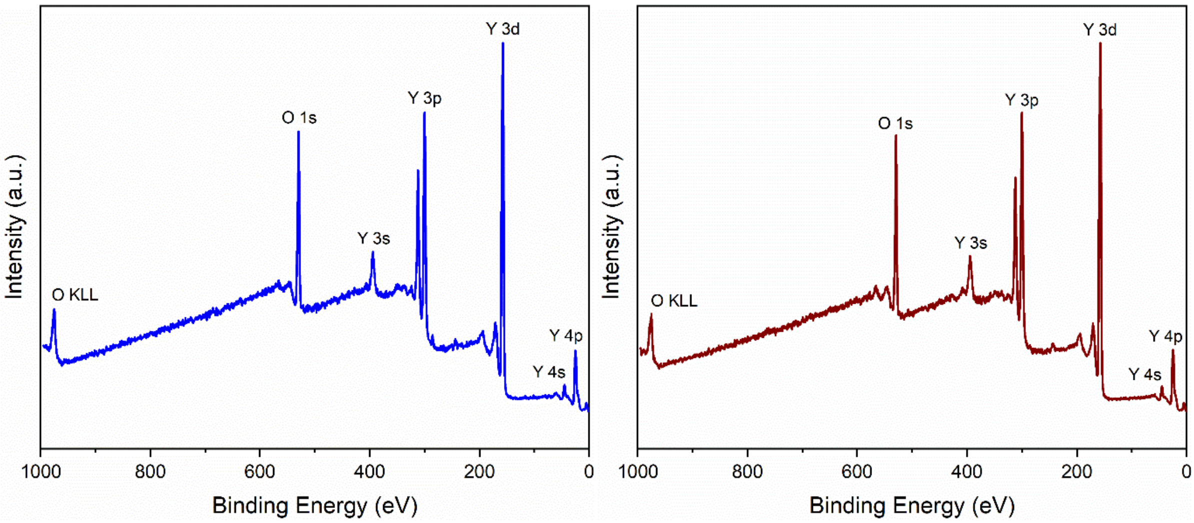

Figure S 3. XPS survey spectra for the as-introduced 40 nm Y_2O_3 thin film on Si(100) deposited at 300 °C (left chart in blue) and for the 1 min Ar^+ sputtered surface (right chart in red).

S6. J-E Characteristics of the MIS Capacitors

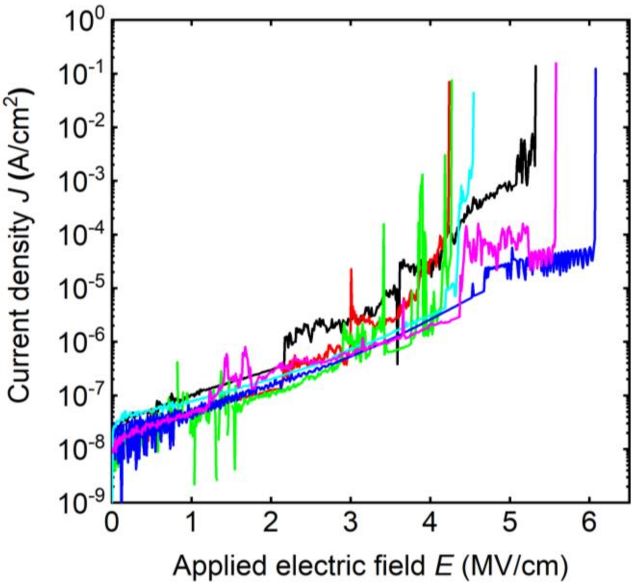

Figure S 4. Leakage current density J as a function of the electric field E for several MIS devices incorporating Y_2O_3 (d = 24 nm) deposited at T = 300 °C. Each color represents a J-E characteristic of an individual device with identical device geometries.

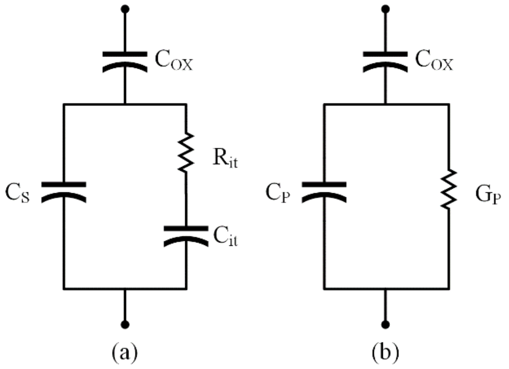

Figure S 5. Equivalent circuit of an MIS capacitor including interface-trap effect (reproduced from Nicollian and Goetzberger).[5]

Equations (1-3):

$$C_p = C_S + \frac{C_{it}}{1+(\omega\tau_{it})^2} \quad (1)$$

$$\frac{G_P}{\omega} = \frac{q\omega\tau_{it}D_{it}}{1+(\omega\tau_{it})^2} \quad (2)$$

$$D_{it} = \frac{2G_{p,max}}{q\omega_{max}} \quad (3)$$

S7. References

[1] P. de Rouffignac, J.-S. Park, R. G. Gordon, *Chem. Mater.* **2005**, *17*, 4808–4814.

[2] A. P. Milanov, R. A. Fischer, A. Devi, *Inorg. Chem.* **2008**, *47*, 11405–11416.

[3] A. P. Milanov, K. Xu, S. Cwik, H. Parala, T. de los Arcos, H.-W. Becker, D. Rogalla, R. Cross, S. Paul, A. Devi, *Dalton Trans.* **2012**, *41*, 13936.

[4] S. Karle, V.-S. Dang, M. Prenzel, D. Rogalla, H.-W. Becker, A. Devi, *Chem. Vap. Depos.* **2015**, *21*, 335–342.

[5] E. H. Nicollian, A. Goetzberger, *Appl. Phys. Lett.* **1965**, *7*, 216–219.
